# The Study of Medicine

**Published:** 1835-01-01

**Authors:** 


*The Study of Medicine.* By John Mason Good, m.d. *Fourth Edition.* By Samuel Cooper, Professor of Surgery in the University of London, &c.—London, 1834. 4 vols. 8vo. pp. 2486.

We recollect that a sprightly contemporary once asserted that Mason Good's Study of Medicine, and Cooper's Surgical Dictionary, were together a sufficient library for a practical man. Now, though we do not go quite so far as this, and hold with the poet, that "Dulcius ex ipso fonte bibuntur aquæ," still we must own that the two works in question will go a long way towards the formation of a small select library, which is to be really read, and vigorously thumbed; and Mason Good's book, in particular, will stand in lieu of scores of those meagre twelve-shilling and nine-shilling, and slim seven-shilling volumes, which, when drawn up in long files, make very big lacunæ in moderate men's purses.

The excellent work before us is so well known, and so highly esteemed, that comment or extract may seem unnecessary; yet it may not be disagreeable to those of our readers who possess one of the earlier copies, if we give a few quotations from Mr. S. Cooper's very judicious additions.

Under the head of Relaxed Uvula, producing the Dysphagia uvulosa of Mason Good, his editor observes,

"The remedy on which Dr. Granville chiefly relies is a gargle containing lunar caustic, the strength of which should be varied, according to the state of the uvula itself, and the nervous irritation in the system. Another useful application, mentioned by the same physician, is a powder composed of equal parts of muriate of ammonia and nitrate of potash, with a quarter of Cayenne pepper. This produces great immediate irritation, followed by copious salivation, and expectoration of thick mucus. The powder should be rubbed on with a camel-hair brush twice or thrice a day. Gargles, made with a proportion of sulphuret of potash, are in common use amongst professional singers, for improving the defect of their voices, connected with relaxation of the uvula; and Dr. Granville thinks the practice justified by analogy; sulphuret of potash being known to have in croup the power of converting the stridulous voice into a deep full tone, and being, in fact, the remedy for which Buonaparte awarded a prize of one thousand Napoleons, during the epidemic croup which raged in Paris in 1812."* (P. 90.)

"*Case of Squinting.* It has been ascertained by experiment, that, in individuals who have a confirmed squint of this kind, one of the eyes is too imperfect to see distinctly. Of this, however, the patient is not always conscious, as was evinced in a young lady, whose case is related by Sir Everard Home. Neither she herself, nor her friends, believed that any defect of the eye existed; and, upon being asked if she saw objects distinctly with her eyes, she

"* *Lancet,* No. 377, pp. 280, 281. According to Dr. Granville, who was for several years physician to the Opera company, the uvula, in deep bass singers, is thick and cornuous, but thin and very pointed in the light, silvery, soprano singers. The observations of M. Bennati leave no doubt that the uvula and soft palate have considerable influence over the modulation of the voice; and he has demonstrated that these organs contract in proportional degrees to the ascent of the several musical notes. (See *Annali Universali* for June, 1830; *Bulletin des Sciences Méd.* for May 1830; and *Lancet,* No. 377.) —Ed."

said, certainly, but that one was stronger than the other. To ascertain the truth of this, he covered the strong eye, and gave her a book to read, when, to her astonishment, she found she could not distinguish a letter, or any other near objects. More distant objects she could see, but not distinctly; when she looked at a bunch of keys in the door of a bookcase about twelve feet from her, she could see the bunch of keys, but could not tell how many there were. The obscurity of vision in one eye, then, is the cause of this common species of squinting, and may occasion this irregularity in the following way. The obscure image being so imperfectly formed in the weak eye as to excite little attention in the mind, the use of the eye, and its uniform direction to the same object with the other, may have been neglected from the beginning; for, as distinct vision was obtained at once by the perfect eye, the end was answered, and therefore there was no necessity for any exertion of the other; or, in the effort to get rid of the confused image, the muscles may have acquired an irregular and unnatural action. Under either of these circumstances, the eye is directed towards the nose, because, as Sir Everard Home remarks, this direction is determined by the superior force of the adductor muscle." (P. 184.)

*Case of Tænia discharged from the Meatus Urinarius.* "The editor, through the kindness of his friend, Mr. Docker, late of Canterbury, has been furnished with the particulars of a young woman, upwards of twenty years of age, who has been under the care of Mr. Law, of Penrith, in Cumberland, since October 1830, and has discharged several thousand portions of tænia from the meatus urinarius. In a letter, dated Penrith, August 29, 1833, Mr. Law states, that 'she first felt a sensation like that of a rupture of the bladder, when in the act of stooping to cut a corn in August 1829. From that time she had discharges of bloody urine occasionally, with the sensation of something moving in the bladder, (more particularly so after each evacuation,) and had concluded that this was a worm. However, no mention was made of this to any one, although her health was impaired, until I was called to attend her for an attack of laryngitis, in October 1830. Blisters being used, brought on retention of urine, with cystitis, which rendered the use of the catheter necessary. After this had been conquered, she mentioned her feelings and apprehensions to me, which for some time I treated as imaginary, till at length, from the greatly disturbed state of the mucous membrane of the bladder, evidenced by deposition in large quantities of a white sediment in the urine, I was led to try the exhibition of Spirit. Terebinth., both by the mouth and injection. Great irritation ensued, but a small portion (about eight joints) of tænia was discharged *per urethram* alive. This led me to the determination to try the effect of opiate solutions, frequently injected into the bladder, which, by keeping the worm constantly under its influence, might destroy it. This, persevered in for three days, answered the purpose: all motion of the worm ceased; and, by an expansion of the urethra, its discharge was effected in large quantities, but in so decayed and broken a state, that its parts could not be numbered; but I am certain that there could not have been less than two thousand joints. With these there was much hemorrhage, also membranes, and other substances. From this date (January 1831,) to the beginning of April of the same year, there were no indications of more tænia; yet the urine was generally tinged with blood, and deposited the white sediment in less quantities than formerly. During this period, an anodyne injection had been used almost daily, as the irritation of the bladder was considerable. At this time she again felt the motion of tæniæ, and I again resorted to frequent opiate injections, but without success, for some days, and then determined upon the administration of Spirit. Terebinth. by the mouth, a teaspoonful of which was taken on the morning of the 18th of April, and which passed by the bladder in an hour and a half, bringing off some portions of the worm in a recent state, with a net-like membrane. From this date to September 20th, there were passed from the bladder *per urethram* 1239 joints, of different sizes, from one third to the eighth of an inch in width, and which were all preserved, with portions of a net-like membrane, and fungi, either like the liver or the muscles of a fowl, and sometimes of a fleshy fibrous substance, having the appearance of the muscular coat of the bladder, with a shaggy surface on one side, resembling the villous coat of the intestines. These were brought away almost daily, while the urine diminished in quantity, rarely exceeding four ounces in twenty-four hours. A pause ensued from the last date to November 16th, of the same year, during which time neither worm nor substances passed off; but the fluid discharged was bloody, and always highly offensive. On this day the Spirit. Terebinth. was again administered, and brought off in an hour two small pieces of net-like cellular tissue, with nine joints of middle-sized worm interwoven with it. From this time to January 18, 1832, there were 773 joints preserved, of different sizes; and these were generally accompanied with membrane and fungi of different kinds. From this date to March 27, there were no portions of tænia passed; but on that day the Sp. Terebinth. was again taken, and, before May 1st, 853 joints passed, making a total of 2865, now in my possession, besides a very small and apparently perfect worm of twenty-nine joints. During the period mentioned there have been at different times profuse hemorrhages from the intestines, surmised to arise from the ascending colon, which have reduced my patient to a very weak state. She is now (August 23) in tolerable health, but again bringing away, from the use of the Spir. Terebinth., more tæniæ. On the 21st instant, there came off four joints of a small worm, with two small fungi.

"Among the singular and unaccountable phenomena accompanying this affection, is the rapidity with which Spirit. Terebinth.

now passes from the stomach to the bladder. It is invariably felt in the bladder in less than twenty seconds from being taken; and its evacuation *per urethram* rarely exceeds two minutes: on one occasion it passed in one minute and a quarter; and, from the recent appearance of the worm and fungi, seems to separate them in passing.

"Mr. Docker is in possession of some specimens of the worm, fungi, and membranes." (Vol. iv. p. 354, *note*.)

After this follows another letter, dated April 15th, 1834, and containing additional particulars of this remarkable case, and tables giving the doses of turpentine administered, and the time when they were felt in the bladder: the period has diminished gradually from twenty-eight minutes to eight or ten seconds after taking the oil.

*Treatment of Porrigo Favosa.* "Two principal objects have generally been kept in view in the treatment of porrigo favosa: in some methods, the cure of the pustular inflammation of the skin, either rationally or empirically, is the main thing aimed at; in others, the evulsion of the hairs constitutes the chief measure. Amongst the former plans, what Rayer terms the antiphlogistic and derivative appears to him the best. The abstraction of blood is seldom necessary. Decoction of linseed, and emollient cataplasms, applied to the shaven scalp, loosen the scales, and diminish the inflammation; but, in order to bring about a cure, Rayer assists these means with two blisters on the arms, from which a discharge is to be maintained for two or three months. But, from the moment that the inflammation has extended to the bulbs of the hair, Rayer sets down every treatment that does not aim at producing either the evulsion or the falling-off of the hair, as ineffectual. For this purpose he prefers the depilatory treatment adopted by MM. Mahon, who pay particular attention to the following objects: 1. To that of cleansing the scalp, and keeping it as much so as possible. 2. To that of separating, without pain, the hairs whose bulbs are inflamed. They begin with cutting the hair, so as to leave it only two inches in length. They then loosen and remove the scabs with hog's lard or a linseed poultice; and afterwards wash the head with soap and water. This plan is continued for four or five days, at the expiration of which the second part of the treatment begins, and a depilatory pomatum is applied to all the affected parts of the scalp every other day. This practice is persisted in for a month, six weeks, or two months. On the days when the pomatum is not used, a fine comb is passed through the hairs, by which means they are detached without pain. At the end of a fortnight, a depilatory powder is sprinkled among the hairs once a week. The next day the fine comb is used, and the depilatory pomatum employed again. In a month or six weeks the first depilatory pomatum is discontinued, and another substituted for it, during a fortnight or a month; after which, the inunctions are made only twice a week, till the redness is completely removed. The comb, which is to be used on the days when the pomatum is not applied, is to be smeared with lard or oil, and only lightly pressed upon. MM. Mahon's depilatory powder and pomatum seem to owe their activity to their containing lime, with a small quantity of subcarbonate of potash, and charcoal. (See Rayer, tom. i. p. 508.) This depilatory treatment, which was deemed unnecessary by Willan and Bateman, is considered indispensable by Rayer; yet, at the Bloomsbury Dispensary, we cure all cases without it. The hair is kept short, the head gently washed or fomented every day, and, as soon as the inflammation has abated, and the scabs have been removed with a little lard, an ointment is applied, consisting of about $3\frac{1}{2}$ grs. of oxymuriate of mercury, ʒj. of white precipitate, and ℥j. of lard. A few grains of subcarbonate of soda and rhubarb, with or without a small proportion of Hydrargyrum cum Creta, according to circumstances, are likewise given once or twice a day.—Ed." (Vol. iv. p. 493, *note.*)

These quotations may serve to show the diligence with which Mr. S. Cooper has performed his arduous duty, and the success which has attended his endeavours to adorn the pages of this valuable text-book with all the modern improvements in the practice of physic.

